# Thermoplastic Mask-Induced Contact Dermatitis: A Case Report

**DOI:** 10.7759/cureus.23815

**Published:** 2022-04-04

**Authors:** Louis Cappelli, Spencer Poiset, Benjamin Greenberger, Voichita Bar-Ad

**Affiliations:** 1 Radiation Oncology, Thomas Jefferson University Hospital, Philadelphia, USA; 2 Radiation Oncology, University of Pittsburgh Medical Center, Hamot, USA

**Keywords:** oncology, head and neck neoplasms, head and neck, contact dermatitis, thermoplastic mask

## Abstract

Thermoplastic masks are commonly used in radiation therapy to immobilize a patient's head and neck during treatment. They are primarily composed of non-toxic polyester compounds that can be manipulated with heat to mold the shape of a patient's head and neck. There is little previously reported evidence of these masks causing allergic contact dermatitis. We present a case of a 44-year-old female with a history of squamous cell carcinoma of the right tonsil with multiple enlarged lymph nodes following surgical excision of the right tonsillar mass and ipsilateral neck dissection elected to undergo adjuvant radiation therapy with volumetric modulated arc therapy (VMAT) technique without concurrent chemotherapy. A thermoplastic mask was issued prior to radiation therapy. Following the mask fitting, the patient developed an allergic contact dermatitis reaction of the head and neck in areas covered by the mask. Her symptoms worsened with continued use of the thermoplastic mask and radiation therapy. As the patient continued and eventually finished the radiation treatment regimen, the dermatologic symptoms failed to respond to topical facial moisturizer and steroid treatment. The contact dermatitis reaction did not completely dissipate until about three months following completion of radiation therapy and contact with the thermoplastic mask. Thermoplastic masks are not known to cause an allergic contact dermatitis reaction. There is only one other reported case documented in the literature. Such reactions can alter the course of radiation therapy if symptoms are severe enough to disrupt treatment or if they cause worsening of the radiation dermatitis. Allergic contact dermatitis to thermoplastic masks should be well documented in the future to better understand the cause and possible risk factors related to the reaction.

## Introduction

Thermoplastic masks are commonly used to immobilize the patient's head while undergoing treatment during radiation therapy to the head and neck area [1]. They are primarily composed of polyester-based materials, predominantly polycaprolactone (PCL) [2]. PCL is not commonly associated with causing allergic contact dermatitis reactions and is considered a non-toxic polyester [3]. There are very few reports of patients experiencing allergic contact dermatitis reactions to thermoplastic masks in the medical literature. In this study, we report a case of allergic contact dermatitis to a thermoplastic mask. 

## Case presentation

A 44-year-old Caucasian female presented for adjuvant radiation therapy (RT) consultation following diagnosis of p16 positive stage II squamous cell carcinoma of the right tonsil (T1N1M0) with multiple involved lymph nodes and subsequent surgical excision of the right tonsillar mass and right neck dissection. Her past medical history was remarkable for Crohn's disease, human leukocyte antigen B27 (HLA-B27) positive ankylosing spondylitis, drug-induced lupus erythematosus from infliximab, multiple basal cell carcinomas treated via Mohs surgeries, and known allergies to penicillins and infliximab. The patient was taking certolizumab for her Crohn's disease, and her drug-induced lupus did not require medical therapy following discontinuation of the infliximab. It was determined that due to the multiple involved lymph nodes identified in the right neck level II and level IIA containing metastatic squamous cell carcinoma, further RT was necessary. After multidisciplinary discussion, concurrent chemotherapy was not determined to be indicated. The RT was applied to the tumor bed and at-risk cervical lymph nodes with a total dose of 60 gray (Gy) in 30 fractions, using 2 Gy per fraction. The patient had no previous RT or chemotherapy exposure.

**Figure 1:**
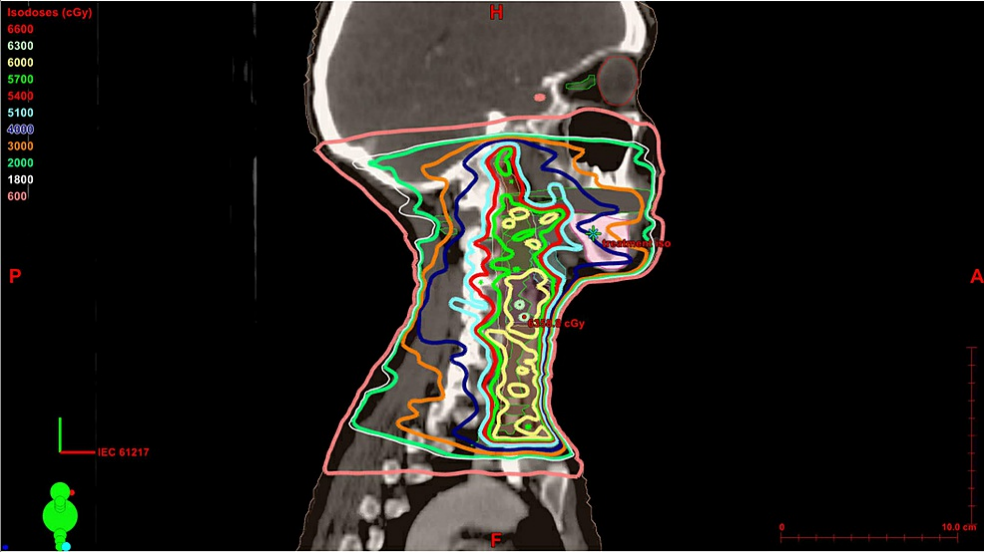
Approved radiation therapy plan

The patient was scheduled for computed tomography (CT) simulation and fitting of Brainlab® thermoplastic mask required for her RT. The approved RT plan (see Figure 1) included cervical lymph node (LN) levels Ib (right), II (bilaterally), II high (right), III (bilaterally), IV (bilaterally), and V (right). The corresponding maximum dose received by each LN level was 65.27 Gy (Ib), 65.64 Gy (II left), 65.02 Gy (II right), 65.11 Gy (II high right), 63.96 Gy (III left), 65.21 Gy (III right), 63.96 Gy (IV left), 64.57 Gy (IV right), and 65.25 Gy (V right). The Brainlab® thermoplastic mask was made seven days prior to the first irradiation. Mask fitting was accomplished by first placing the mask in warm at 160 degrees Fahrenheit for 15 minutes and then spraying the mask with water to cool it down before conforming it to the patient's head and neck.

**Figure 2:**
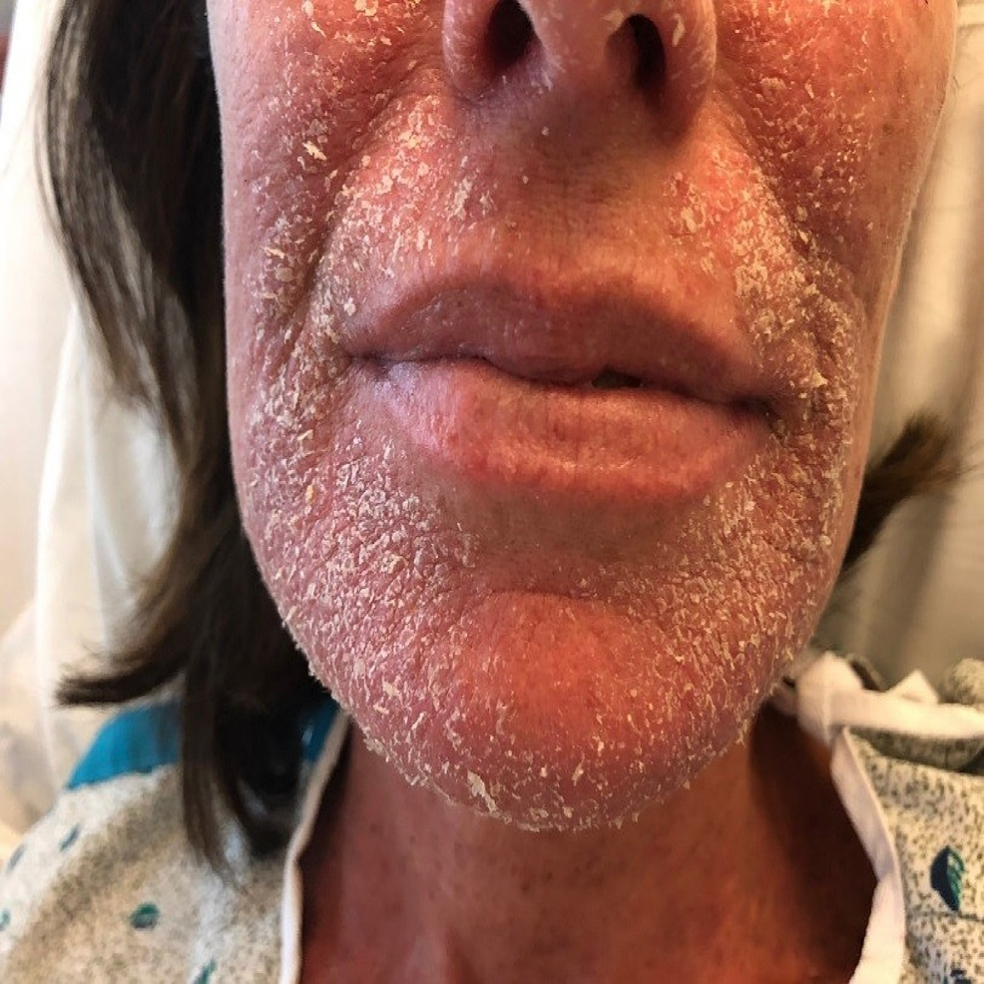
Perioral contact dermatitis rash

**Figure 3:**
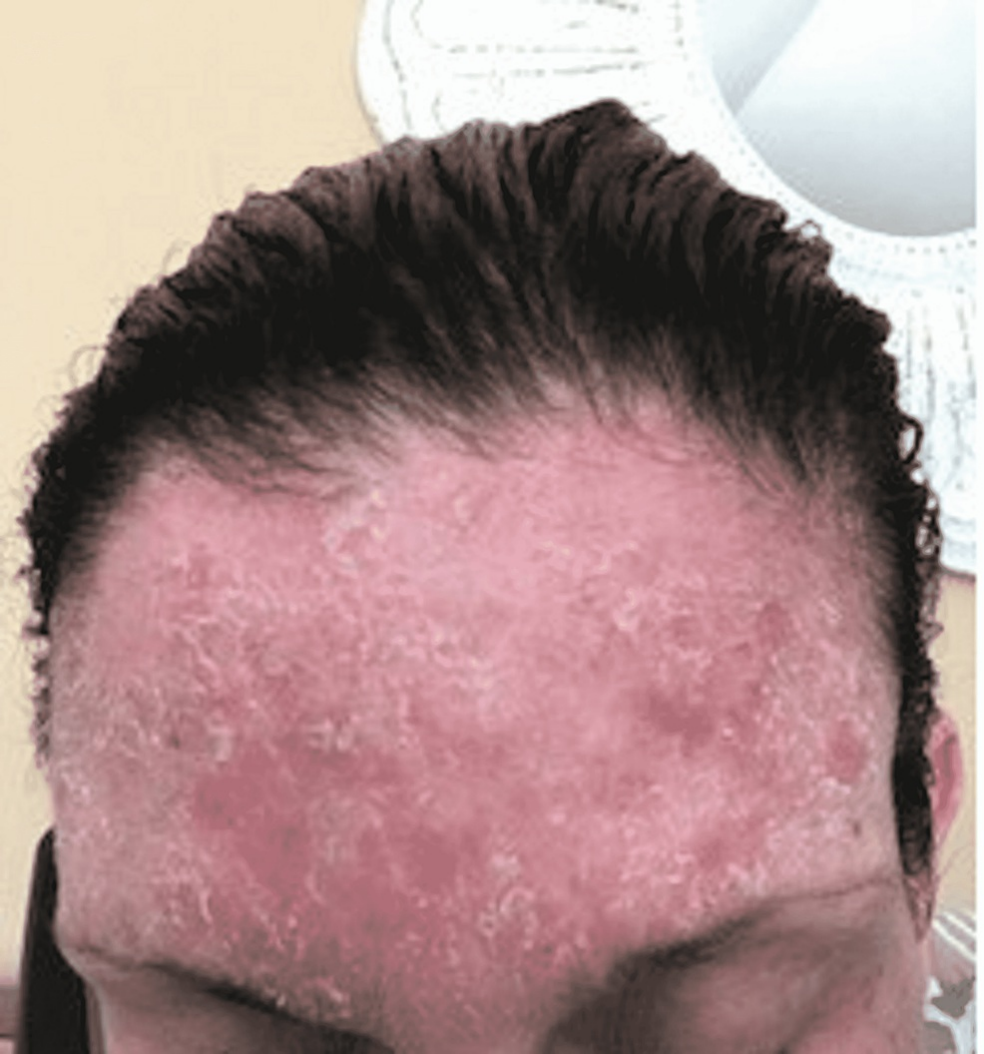
Forehead contact dermatitis rash

She was then seen for radiation therapy treatment one week following the CT simulation and thermoplastic mask fitting. At this time, she presented with a mild erythematous facial rash around her mouth and nose without skin breakdown or drainage. The patient was provided a low dose topical steroid triamcinolone 0.1% and petroleum jelly facial moisturizer according to the dermatologist's suggestion for management of the dermatologic reaction. Over the next two weeks, the patient continued radiation therapy treatment with thermoplastic mask exposure. The rash persisted and became more erythematous and more painful despite daily topical management (Figure 2). The patient was then advised to undergo a shave biopsy of the erythematous perioral and paranasal rash, which was taken approximately one month following onset. The impression from the histopathological examination suggested allergic contact dermatitis, likely from the thermoplastic mask used for her radiation therapy. The rash area was consistent with areas where portions of the thermoplastic mask contacted the patient's skin. The erythematous rash continued to worsen over the course of her RT and further thermoplastic mask exposure despite topical steroid and daily petroleum jelly facial moisturizer treatment. It expanded down to the patient's anterior neck and forehead without skin breakdown or drainage (Figure 3). There was also associated swelling and tenderness that continued to develop and worsen with the rash as it progressed throughout the radiation therapy treatment regimen. 

The rash was described by dermatology as a confluent scaly red rash in the distribution of the radiation mask that resembled the patient's previous cutaneous skin rash from lupus but was not responsive to the topical steroid treatment. A shave biopsy of skin measuring 0.4 x 0.3 x 0.1 cm was taken. Histopathological findings of the specimen were described as spongiotic psoriasiform hyperplasia accompanied by superficial perivascular infiltrate of lymphocytes and eosinophils. Diagnosis from the pathology sample indicated allergic contact dermatitis.

Mild improvement in her skin condition was noticed primarily in the neck region with continued topical steroid and moisturizer treatment two weeks post-RT. The forehead, perioral, and paranasal distribution of the rash remained with its concurrent irritation. Segments of the remaining rash actually worsened one month following radiation therapy with increased erythema, dryness, and irritation while applying the current treatment regimen. Notable reduction in rash presentation and symptoms were not seen until seven weeks post thermoplastic mask exposure and RT. Complete resolution of the rash and symptoms were not seen until approximately twelve weeks following RT and thermoplastic mask exposure with continued daily topical steroid and facial moisturizer regimen.

## Discussion

Thermoplastic masks have allowed for improved immobilization of the head and neck region to allow for more precise radiation therapy treatment of head and neck cancers. They play an important role in ensuring the accuracy and precision of delivering radiation treatment by minimizing patient movement, as well as decreasing setup variability between daily treatments [1,4,5]. Without the positional reproducibility to limit day-to-day setup variation during the entire radiation treatment delivery process, which typically lasts around 6-7 weeks, patients are at an increased risk of receiving radiation doses to undesired or non-target areas [5].

Thermoplastic masks used for immobilization in head and neck radiation therapy largely consist of synthetic polymers such as polycaprolactone (PCL) [2]. There have been few documented cases of allergic contact dermatitis reaction to PCL recorded in the literature, especially pertaining to its composition in a thermoplastic mask. Only one other published case report was found describing an acute allergic contact dermatitis reaction to a thermoplastic mask used for immobilization during radiation therapy. Massager et al. published a case report in 2018 where a patient developed an allergic contact dermatitis reaction to a thermoplastic mask. This patient was treated conservatively with antihistamines and topical corticosteroids until the end of the radiation treatment regimen [6]. There are a few other allergic contact dermatitis reactions to PCL further found in the literature, although they are not related to the use of a thermoplastic mask. For instance, Clemmensen et al. reported a case where an allergic contact dermatitis reaction was caused by a cosmetic retinol ester composed of retinyl palmitate in PCL [7]. PCL is commonly used in cosmetics to increase skin penetration of certain compounds such as nanoparticles [8].

Allergic contact dermatitis is a type IV, delayed-type hypersensitivity reaction mediated by the activation of antigen-specific T cells. The sensitized T cells are primarily the T helper 1 (TH1) type. They are sensitized in lymph nodes after coming in contact with the antigen/allergen taken up by Langerhan and dermal dendritic cells. Following sensitization, TH1 cells proliferate and enter circulation. Once re-exposure to the specific allergen/antigen occurs, the TH1 cells activate and release cytokines inducing a local inflammatory response [9]. It is speculated that our patient developed sensitization to PCL or alike polymer in the thermoplastic mask that resulted in a type IV hypersensitivity reaction. Although to confirm this requires further patch testing with PCL polymers.

Since allergic contact dermatitis to PCL is uncommon, other comorbidities in our patient were evaluated to possibly cause an increase in susceptibility to generating an allergic contact dermatitis response. To our surprise, a study in 2007 by Engkilde et al. actually demonstrated an inverse relationship between patients with Crohn’s disease and contact dermatitis. The odds ratio for contact dermatitis in this population was 0.42 (95% CI: 0.23-0.76) and a p-value of 0.004 [10,11]. In addition, there are no studies found in the literature correlating ankylosing spondylitis with allergic contact dermatitis.

Acute radiation-induced dermatitis was considered as the differential for this new onset rash but was concluded to be unlikely as the rash did not appear to follow a dose-dependent distribution and its onset followed the CT simulation before the RT was even started. The defined erythematous nature of the rash in this patient is commonly associated with a cumulative radiation dose of 12-20 Gy and is typically not seen until 2-3 weeks following radiation treatment [12]. Our patient started experiencing symptoms following the thermoplastic mask fitting and within the first week of radiation therapy treatment. In addition, the patient presented to her first post-RT visit with the dermatitis reaction after only being exposed to 6 Gy total in 2 Gy daily fractions. This is not considered a sufficient enough radiation dose to cause the dermatologic symptoms of profound erythema the patient presented with. Moreover, the rash affected areas outside of the treatment field, as seen in the figures, including the forehead. This makes the dermatitis reaction unlikely to be caused by radiation due to the low radiation dose exposure at the time of symptomatic onset as well as its occurrence outside of the prescribed treatment field.

The complications of the case described are significant and can have serious implications for altering the effectiveness of radiation therapy if the dermatitis is severe enough to prevent further treatment or if the typical radiation dermatitis is worsened. Consequences of missed or delayed radiation treatments are well documented and are shown to be associated with an increased risk of cancer recurrence [13].

## Conclusions

In conclusion, we present a rare case of acute allergic contact dermatitis to a Brainlab® thermoplastic mask commonly used for immobilization in head and neck radiation therapy treatment. Dermatitis of the head and neck region during radiation therapy treatment can lead to a significant alteration in the radiation treatment regimen. Future suspected cases of allergic contact dermatitis to thermoplastic masks should be reported with allergic contact dermatitis patch testing results if possible.
